# Swiprosin-1 Negatively Regulates Osteoclast Differentiation and Bone Resorption via Akt/MAPK/NF-κB Pathway and αvβ3 Integrin-Dependent Signaling

**DOI:** 10.3390/ijms26178613

**Published:** 2025-09-04

**Authors:** Yoon-Hee Cheon, Sung Chul Kwak, Chong Hyuk Chung, Chang Hoon Lee, Myeung Su Lee, Ju-Young Kim

**Affiliations:** 1 Musculoskeletal and Immune Disease Research Institute, School of Medicine, Wonkwang University, Iksan 54538, Republic of Korea; hanleuni@naver.com (Y.-H.C.); ksc960@naver.com (S.C.K.); taylorchung@hanmail.net (C.H.C.); lch110@wku.ac.kr (C.H.L.); 2 Division of Rheumatology, Department of Internal Medicine, Wonkwang University Hospital, Iksan 54538, Republic of Korea

**Keywords:** swiprosin-1 (SWS1)/EFhd2, osteoclast differentiation, F-actin cytoskeleton formation, bone resorption, bone diseases

## Abstract

Swiprosin-1 (SWS1/EFhd2) is a calcium-binding adaptor protein involved in cytoskeletal regulation, but its physiological role in bone homeostasis remains largely undefined. To elucidate its function in osteoclast biology, we examined SWS1 expression and activity during osteoclastogenesis using primary murine bone marrow-derived macrophages, siRNA-mediated knockdown, and SWS1 knockout (KO) mice. SWS1 was predominantly localized to the nucleus in precursor cells and redistributed to the F-actin ring in mature osteoclasts. Receptor activator of nuclear factor-kappa B ligand stimulation significantly downregulated SWS1 mRNA expression. Loss of SWS1 enhanced osteoclast formation, F-actin ring integrity, and bone resorption, accompanied by elevated expression of osteoclastogenic markers. In vivo, male SWS1 KO mice exhibited deteriorated trabecular bone microarchitecture with increased osteoclast numbers. Mechanistically, SWS1 deficiency intensified αvβ3 integrin-associated cytoskeletal signaling and upregulated Akt, MAPK, NF-κB, and PLCγ2 pathways. These results indicate that SWS1 negatively regulates osteoclast differentiation and function by restraining cytoskeletal reorganization and downstream signaling. Collectively, our findings establish SWS1 as a novel modulator of osteoclast activity and a potential therapeutic target for osteolytic bone disorders.

## 1. Introduction

Bone is a dynamic living tissue that undergoes constant remodeling throughout life; bone remodeling is essential for maintaining structural integrity and mineral homeostasis, thereby protecting the body from mechanical stress and serving as a reservoir for calcium [1]. Bone also regulates blood calcium levels and mediates hormonal actions in the intestine, kidney, and skeletal tissues [2]. Healthy bone remodeling is achieved through a delicate balance between bone formation by osteoblasts and bone resorption by osteoclasts [1]. Disruption of this balance results in metabolic bone diseases such as osteoporosis, characterized by excessive bone resorption, or osteopetrosis, which is caused by defective osteoclast activity [2].

Osteoclasts are multinucleated giant cells derived from hematopoietic stem cells that play a central role in bone resorption by degrading the mineralized matrix. Their differentiation and activation are tightly regulated by signals from the bone marrow microenvironment. Among them, receptor activator of nuclear factor-kappa B (NF-κB) (RANK) and its ligand RANKL serve as the primary regulators [2]. The binding of RANK-RANKL directly recruits tumor necrosis factor receptor-associated factors (TRAFs), initiating a cascade of downstream signaling events such as mitogen-activated protein kinases (MAPKs; p-38, ERK, and JNK), NF-κB, and activator protein-1 [3,4,5]. These pathways ultimately lead to the induction of nuclear factor of activated T cells cytoplasmic 1 (NFATc1), the master transcription factor of osteoclastogenesis, along with other osteoclast-specific genes such as osteoclast-associated receptor (OSCAR), β3-integrin, v-ATPase subunit d2 (Atp6v0d2), and cathepsin K (Ctsk) [5,6,7,8,9]. In parallel with RANK signaling, ITAM adaptors (DAP12/FcRγ) activate the Syk–Btk–PLCγ2–Ca^2+^ axis that drives calcineurin–NFATc1 autoamplification, while αvβ3–integrin–Src–cortactin signaling orchestrates actin-ring/podosome assembly required for resorption. In such a network, Ca^2+^ sensors/adaptors are poised to tune both transcriptional and cytoskeletal programs of osteoclasts.

Given this architecture, we considered whether Swiprosin-1 (SWS1), an EF-hand domain-containing protein 2 (EFhd2), might couple ITAM-dependent Ca^2+^ signals to integrin–Src–cortactin-driven cytoskeletal remodeling in osteoclasts. SWS1 was first identified in human CD4^+^ and CD8^+^ T lymphocytes [10]. It is now known to be widely expressed in immune and non-immune cells, including B cells, macrophages, mast cells, epithelial cells, endothelial cells, and neurons [11]. SWS1 is involved in diverse biological processes such as apoptosis, Ca^2+^ signaling, actin cytoskeleton remodeling, cell invasion, and migration, highlighting its multifunctional role in cellular signaling [12,13,14,15,16]. For instance, Zhang et al. demonstrated that SWS1 deficiency impairs macrophage recruitment and cytokine production in a lipopolysaccharide (LPS)-induced sepsis model, suggesting its role in immune modulation and actin dynamics [17].

Structurally, SWS1 harbors two EF-hand Ca^2+^-binding motifs, a proline-rich region capable of engaging Src homology 3 (SH3)-domain–containing partners, and a C-terminal coiled-coil, consistent with roles in Ca^2+^ signaling and protein–protein interactions [18]. As precedents for adaptor/scaffold control of osteoclast programs, calmodulin-dependent Ca^2+^ pathways and the nuclear scaffold SP100 have been implicated in modulating NFATc1-driven transcription, alongside core modules such as DAP12/FcRγ–Syk–Btk–PLCγ2 and integrin–Src–cortactin [19,20,21]. These considerations led us to hypothesize that SWS1 functionally interfaces with proximal RANKL/ITAM signaling and adhesion-coupled cytoskeletal pathways in osteoclasts. Accordingly, we posited that SWS1 restrains osteoclastogenesis by dampening proximal RANKL–TRAF6–MAPK/NF-κB and ITAM-driven Ca^2+^/calcineurin–NFATc1 signaling while limiting αvβ3–integrin–Src–cortactin-dependent actin-ring maturation. This framework provided a direct mechanistic rationale to test SWS1 in osteoclasts in vitro and in vivo.

Therefore, in this study, we investigated the role of SWS1 in osteoclast differentiation and function using both in vitro (small interfering RNA (siRNA)-mediated knockdown and recombinant protein treatment) and in vivo (SWS1-deficient mice) models. Our results reveal a novel function of SWS1 as a negative regulator of osteoclastogenesis and bone resorption.

## 2. Results

### 2.1. SWS1 Is Downregulated During Osteoclastogenesis and Suppresses Osteoclast Differentiation and Function

**Figure 1:**
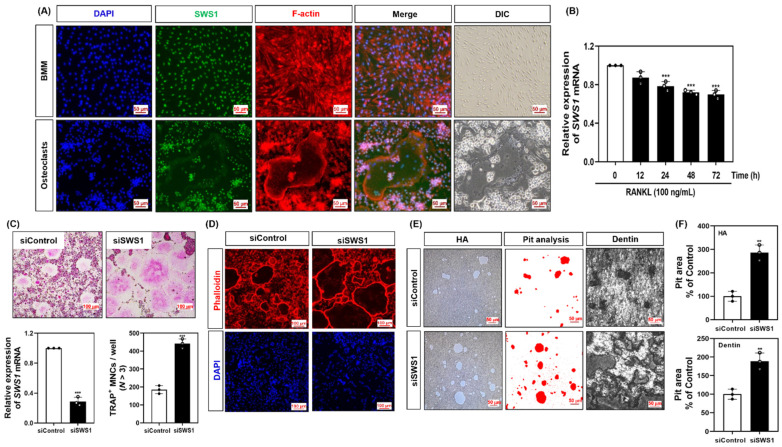
Expression pattern of SWS1 and the effect of SWS1 knockdown on osteoclast differentiation, cytoskeletal organization, and bone resorption. (**A**) Immunofluorescence images showing subcellular localization of SWS1 in BMMs and mature osteoclasts. (**B**) Time-course analysis of SWS1 mRNA expression during RANKL-induced osteoclast differentiation. (**C**) Knockdown efficiency of SWS1 by siRNA and quantification of TRAP-positive multinucleated cells (MNCs). (**D**) F-actin ring staining after SWS1 knockdown. (**E**) Representative images of pit formation on hydroxyapatite (HA)-coated plates and dentin slices following SWS1 knockdown. (**F**) Quantification of resorbed pit area in osteoclasts transfected with siControl or siSWS1. Data are presented as the mean ± SD of three independent experiments. ** *p* < 0.05; *** *p* < 0.001 versus the control or siControl.

To investigate the role of SWS1 in osteoclastogenesis, we first examined its subcellular localization. In undifferentiated bone marrow-derived macrophages (BMMs), SWS1 was predominantly localized in the nucleus. Upon RANKL-induced differentiation, SWS1 was redistributed to the cytoplasm and exhibited a broad, gauze-like distribution within the F-actin belt region of mature osteoclasts, rather than direct co-localization with F-actin (Figure 1A). We next assessed the temporal regulation of SWS1 expression. RANKL stimulation led to a significant, time-dependent downregulation of SWS1 mRNA during osteoclast differentiation (Figure 1B). To evaluate the functional relevance of SWS1, siRNA-mediated knockdown was performed in BMMs. Efficient SWS1 silencing resulted in a marked increase in the number of TRAP-positive multinucleated cells (MNCs), as well as enhanced formation of mature osteoclasts (Figure 1C). Phalloidin staining revealed that siRNA for SWS1 (siSWS1)-treated cells developed more defined and extensive F-actin rings (Figure 1D). Furthermore, bone resorption activity, assessed using hydroxyapatite and dentin surfaces, was significantly elevated in the siSWS1 group compared to controls (Figure 1E).

Together, these findings indicate that SWS1 is negatively regulated during osteoclastogenesis and acts as a suppressor of osteoclast differentiation and function, potentially through modulation of actin cytoskeletal organization.

### 2.2. SWS1 Knockdown Enhances RANKL-Induced Signaling and Osteoclast-Related Gene Expression

**Figure 2:**
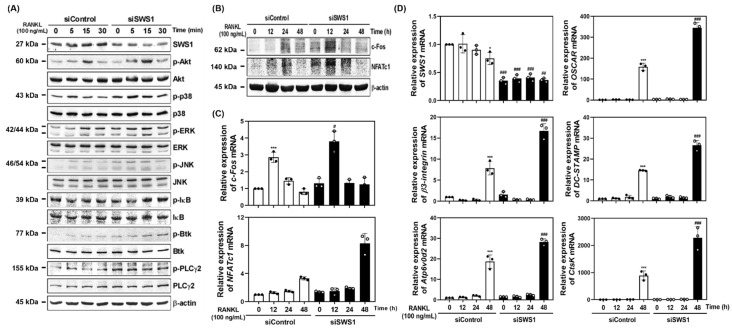
Effect of SWS1 knockdown on RANKL-induced signaling pathways and osteoclast-specific gene expression. (**A**) BMMs transfected with siRNAs were serum-starved for 3 h and exposed to RANKL (100 ng/mL). Immunoblotting for phosphorylated Akt, p38, ERK, JNK, IκB, Btk, and PLCγ2 in SWS1-silenced BMMs. β-actin was used as a loading control. (**B**) Time-course analysis of c-Fos and NFATc1 protein expression. (**C**) Quantitative real-time PCR (qRT-PCR) analysis of c-Fos and NFATc1 mRNA expression. (**D**) Expression of osteoclast-related genes, including SWS1, OSCAR, β3-integrin, DC-STAMP, Atp6v0d2, and Ctsk. The mRNA expression was normalized with *GAPDH*. Data are presented as the mean ± SD of three independent experiments. * *p* < 0.05; *** *p* < 0.001 versus siControl cells. ^#^ *p* < 0.05; ^##^ *p* < 0.01; ^###^ *p* < 0.001 versus the siControl cells at the indicated time point.

To investigate the mechanism by which SWS1 regulates osteoclastogenesis, we examined RANKL-induced intracellular signaling pathways in SWS1-deficient cells. Western blot analysis demonstrated that siRNA-mediated knockdown of SWS1 enhanced the phosphorylation of multiple signaling molecules downstream of RANK, including Akt, p38, ERK, JNK, IκB, Btk, and PLCγ2 (Figure 2A and Figure S3A). These results indicate that SWS1 negatively regulates early osteoclastogenic signaling cascades. We next analyzed the expression of c-Fos and NFATc1, key transcriptional regulators of osteoclastogenesis. In siSWS1-transfected cells, c-Fos protein expression peaked at 12 h post-RANKL stimulation, showing markedly higher levels than the siControl group (Figure 2B and Figure S3B). Total NFATc1 protein increased at 12–24 h in siSWS1-treated cells, consistent with enhanced NFATc1 induction/auto-amplification during osteoclastogenesis; however, NFATc1 transcriptional activity is governed by calcineurin-dependent dephosphorylation and nuclear translocation rather than total abundance. Consistently, real-time RT-PCR analysis revealed that c-Fos mRNA expression was significantly increased at 12 h in response to RANKL and was further upregulated in the siSWS1 group (Figure 2C). In contrast, NFATc1 mRNA expression showed a pronounced increase at 48 h following RANKL treatment in siSWS1 cells, indicating temporally distinct transcriptional regulation of these two master regulators. Furthermore, osteoclast-related genes involved in cell fusion and function—including OSCAR, β3-integrin, DC-STAMP, Atp6v0d2, and Ctsk—were all significantly upregulated by siSWS1 treatment, particularly at 24–48 h post-RANKL stimulation (Figure 2D). These findings suggest that SWS1 deficiency amplifies both early signaling cascades and late-stage gene expression programs, thereby enhancing osteoclast differentiation and activation.

### 2.3. SWS1 Deficiency Promotes Osteoclast Hyperactivation and Trabecular Bone Loss In Vivo and Ex Vivo

**Figure 3:**
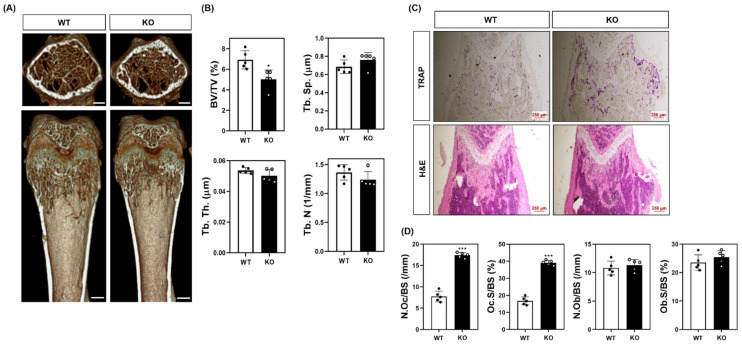
Bone phenotypes in WT and SWS1 KO mice. (**A**) Representative micro-CT images of distal femurs (scale bar = 0.5 mm). (**B**) Comparison of bone mineral density (BMD, g/cm^2^), bone volume/total volume (BV/TV, %), bone surface (mm^2^), trabecular number (1/mm), trabecular thickness (mm), and trabecular separation (mm) at 5 weeks in mice femurs. (**C**) TRAP and H&E staining of the proximal femur. (**D**) Quantification of osteoclast number per bone surface (N.Oc/BS), osteoclast surface per bone surface (Oc.S/BS), osteoblast number per bone surface (N.Ob/BS), and osteoblast surface per bone surface (Ob.S/BS). Data are presented as the mean ± SD of three independent experiments. * *p*< 0.05; *** *p*< 0.001 versus the SWS1 WT.

**Figure 4:**
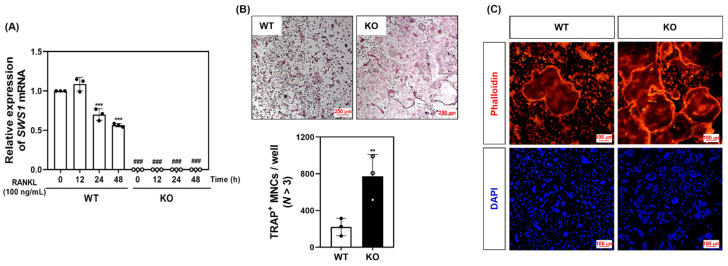
Osteoclast differentiation and cytoskeletal structure in WT and SWS1 KO BMMs. (**A**) qRT-PCR analysis of SWS1 mRNA expression during osteoclast differentiation. (**B**) TRAP staining and quantification of multinucleated osteoclasts. (**C**) F-actin ring staining in differentiated osteoclasts. Data are presented as the mean ± SD of three independent experiments. ** *p* < 0.01; *** *p* < 0.001 versus siControl cells. ^###^ *p* < 0.001 versus the SWS1 WT at the indicated time point.

**Figure 5:**
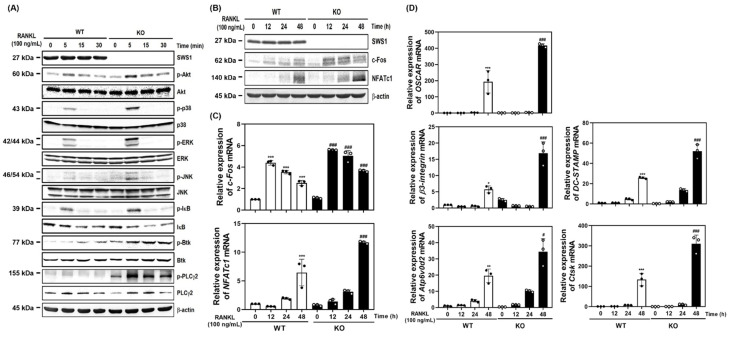
Activation of osteoclastogenic signaling and gene expression in WT and SWS1 KO BMMs. (**A**) Immunoblotting of phosphorylated signaling molecules (Akt, MAPKs, IκB, Btk, and PLCγ2). (**B**) Western blot analysis of c-Fos and NFATc1 protein expression. (**C**) qRT-PCR analysis of c-Fos and NFATc1 mRNA levels. (**D**) mRNA expression levels of osteoclast-specific genes. Data are presented as the mean ± SD of three independent experiments. * *p* < 0.05; ** *p* < 0.01; *** *p* < 0.001 versus SWS1 WT cells. ^#^ *p* < 0.05; ^###^ *p* < 0.001 versus SWS1 WT cells at the indicated time point.

To evaluate the physiological relevance of SWS1 in bone remodeling, we analyzed the skeletal phenotype of SWS1 knockout (KO) mice. Micro-CT imaging of distal femora revealed a marked reduction in trabecular bone volume in KO mice compared to wild-type (WT) controls (Figure 3A). Quantitative analysis showed a significant decrease in bone volume fraction (BV/TV), accompanied by a mild increase in trabecular separation (Tb.Sp), while trabecular thickness (Tb.Th) and number (Tb.N) remained unchanged (Figure 3B). Histological evaluation further supported these findings: TRAP staining of femoral sections showed an increased number of TRAP-positive osteoclasts along the bone surface, and H&E staining demonstrated expanded marrow spaces in KO mice (Figure 3C). Morphometric analysis confirmed a significant increase in both osteoclast number per bone surface (N.Oc/BS) and osteoclast surface per bone surface (Oc.S/BS) in KO animals (Figure 3D), indicating elevated osteoclast activity and consequent bone loss in vivo. By contrast, baseline osteoblast metrics—osteoblast surface per bone surface (Ob.S/BS) and osteoblast number per bone surface (N.Ob/BS)—were comparable between genotypes (Figure 3D). Consistently, primary osteoblast cultures showed unchanged ALP staining/activity and Alizarin Red S staining/activity, and von Kossa staining of femora revealed no overt differences (Figure S7). Together, these data suggest that SWS1 deficiency predominantly impacts the osteoclast arm of bone remodeling under homeostatic conditions. To determine whether this phenotype arises from intrinsic changes in osteoclast precursors, we next assessed osteoclast differentiation ex vivo. BMMs isolated from KO mice lacked SWS1 expression and showed significantly enhanced osteoclastogenesis upon RANKL stimulation, as evidenced by increased TRAP^+^ multinucleated cell formation and more extensive F-actin ring structures (Figure 4A–C). These results indicate that SWS1 intrinsically suppresses osteoclast differentiation and cytoskeletal maturation. To explore the underlying molecular mechanisms, we examined RANKL-induced signaling and transcriptional responses. Compared to WT cells, KO BMMs exhibited stronger and more sustained phosphorylation of key signaling molecules, including Akt, p38, ERK, JNK, IκB, Btk, and PLCγ2 (Figure 5A and Figure S4A). This was accompanied by increased expression of c-Fos protein (elevated at 12–24 h) and NFATc1 protein (peaking at 48 h) in KO cultures (Figure 5B and Figure S4B). Consistent with this, c-Fos mRNA was significantly upregulated at 12–48 h, and NFATc1 mRNA at 48 h post-RANKL stimulation in KO cells (Figure 5C). Additionally, osteoclast-related functional genes—*Oscar*, *β3-integrin*, *DC-STAMP*, *Atp6v0d2*, and *Ctsk*—were robustly induced in KO BMMs, particularly at 48 h (Figure 5D). Together, these in vivo and ex vivo results demonstrate that SWS1 functions as a key negative regulator of osteoclast differentiation and activation. Its deficiency leads to hyperactive osteoclastogenesis and pathological bone loss, mediated through amplified RANKL signaling and upregulation of osteoclastogenic gene programs.

### 2.4. SWS1 Modulates Osteoclast Cytoskeletal Signaling via the Src–Syk–Cbl–Cortactin Pathway

**Figure 6:**
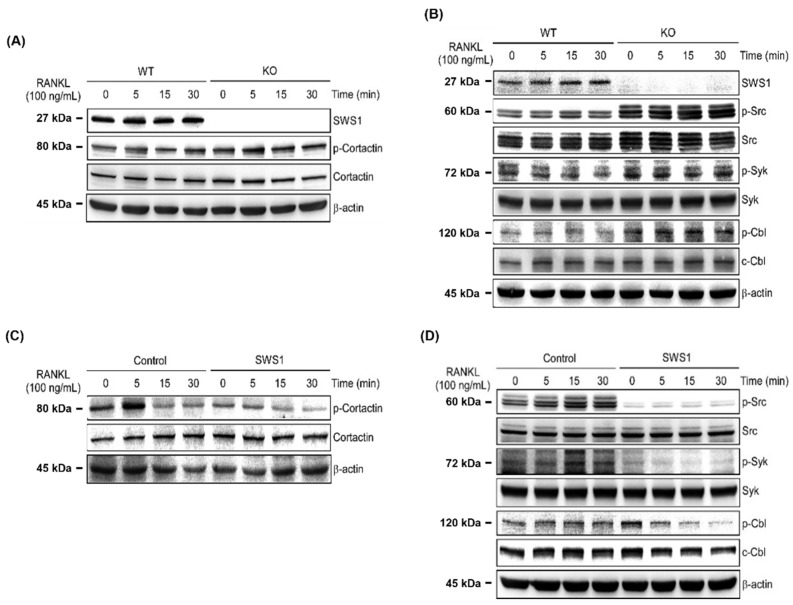
Regulation of cytoskeletal signaling by SWS1 in WT and KO osteoclast precursors. (**A**) Western blot analysis of cortactin phosphorylation. (**B**) Immunoblotting of phosphorylated Src, Syk, and Cbl. (**C**) Cortactin phosphorylation in BMMs treated with recombinant SWS1 protein. (**D**) Phosphorylation status of Src, Syk, and Cbl following recombinant SWS1 treatment.

To explore how SWS1 regulates osteoclast cytoskeletal organization and activation, we examined key components of the integrin-associated signaling cascade. In BMMs from SWS1 KO mice, phosphorylation of cortactin—a downstream effector of actin cytoskeleton remodeling—was markedly enhanced following RANKL stimulation compared to WT cells (Figure 6A and Figure S5A). Similarly, KO BMMs exhibited increased phosphorylation of Src, Syk, and Cbl proteins upon RANKL treatment, indicating hyperactivation of integrin-mediated signaling in the absence of SWS1 (Figure 6B and Figure S5B). To confirm the regulatory role of SWS1 in this pathway, we treated WT BMMs with recombinant SWS1 protein and assessed its effect on the same signaling molecules. SWS1 treatment suppressed RANKL-induced phosphorylation of cortactin in a time-dependent manner (Figure 6C and Figure S5C). In addition, phosphorylation of Src, Syk, and Cbl was significantly attenuated in the presence of recombinant SWS1 compared to control-treated cells (Figure 6D and Figure S5D). These findings suggest that SWS1 negatively regulates osteoclast cytoskeletal signaling by inhibiting activation of the Src–Syk–Cbl–cortactin axis, thereby limiting cytoskeletal maturation and bone-resorptive function during osteoclastogenesis.

## 3. Discussion

Osteoclasts are highly specialized, multinucleated cells responsible for bone resorption, and their activity is tightly regulated to maintain skeletal homeostasis. Dysregulation of osteoclastogenesis leads to pathological bone loss, as seen in osteoporosis, inflammatory bone diseases, and certain cancers [22]. Although the molecular mechanisms governing osteoclast differentiation have been extensively studied—particularly RANKL-induced activation of MAPKs, NF-κB, and calcium signaling—the role of adaptor and scaffold proteins in fine-tuning these pathways remains incompletely understood.

In this study, we identified SWS1/EFhd2 as a novel negative regulator of osteoclast differentiation and function. Previous studies have characterized SWS1 as a cytoskeletal Ca^2+^-binding protein with roles in immune cell signaling, B cell receptor organization, and actin remodeling [13,14,15,16,17,18]. However, its function in bone metabolism has not been explored. Our data establish that SWS1 constrains osteoclastogenesis through multiple signaling checkpoints, both upstream and downstream of RANKL-RANK signaling. A key finding of this study is the dynamic downregulation of SWS1 expression during RANKL-induced differentiation of bone marrow-derived macrophages. This suggests that SWS1 may act as a molecular brake that is actively removed to allow osteoclast maturation. Consistent with this idea, SWS1 deficiency—via siRNA or genetic KO—led to enhanced osteoclast formation, increased F-actin ring organization, and elevated bone-resorbing activity. Notably, the opposite phenotype was observed with recombinant SWS1 treatment (Figures S1, S2 and S6), which significantly suppressed osteoclast differentiation and cytoskeletal maturation, further confirming its inhibitory role. Mechanistically, our study reveals that SWS1 negatively regulates both canonical and non-canonical osteoclastogenic signaling. In the early phases of differentiation, SWS1 deficiency potentiated RANKL-induced phosphorylation of p38, ERK, JNK, Akt, and IκB, consistent with enhanced activation of the MAPK and NF-κB pathways—key regulators of *c-Fos* and *NFATc1* transcription [23,24,25,26]. This aligns with previous models in which activation of these pathways promotes the expression of osteoclast-specific genes, including *Oscar*, *DC-STAMP*, and *Ctsk* [26,27]. Notably, the increase in total NFATc1 reflects enhanced induction/auto-amplification during osteoclastogenesis, whereas its transcriptional activity is governed by calcineurin-dependent dephosphorylation and nuclear translocation rather than protein abundance per se.

Importantly, SWS1 also modulated co-stimulatory signaling cascades mediated by ITAM adaptors, including Syk, Btk, and PLCγ2. These molecules coordinate calcium signaling and contribute to the autoamplification of NFATc1, the master transcription factor of osteoclastogenesis [23,28]. Accordingly, SWS1 loss may accelerate the induction arm (NFATc1 upregulation) downstream of RANKL/ITAM–Ca^2+^ inputs, while functional activation ultimately requires calcineurin-mediated dephosphorylation and nuclear import of NFATc1. Our findings suggest that SWS1 suppresses this axis, as shown by enhanced phosphorylation of ITAM-pathway components in SWS1-deficient cells and the opposing suppression in SWS1-treated cells. This identifies SWS1 as a key intracellular modulator of calcium signaling in osteoclast precursors, expanding on its previously reported roles in B cell calcium flux regulation [13]. Beyond transcriptional regulation, we provide compelling evidence that SWS1 governs cytoskeletal remodeling in osteoclasts. The integrity of the actin ring and podosome belt is essential for bone adhesion and resorption, processes driven by integrin–Src–Cbl–cortactin signaling complexes [29,30,31,32]. SWS1-deficient BMMs exhibited increased phosphorylation of Src, Syk, Cbl, and cortactin, indicating hyperactivation of this cytoskeletal machinery. Recombinant SWS1 treatment reversed these changes, suggesting that SWS1 modulates integrin-associated signaling and actin ring formation. Given that SWS1 contains an SH3 domain, it may potentially interfere with Src–integrin interactions, although direct binding remains to be confirmed in future studies.

Beyond SWS1, multiple classes of regulators shape osteoclastogenesis and resorption. The secreted Wnt antagonist Sfrp4 can restrain osteoclast differentiation cell-autonomously by repressing Ror2–JNK signaling while also influencing cortical/trabecular remodeling in vivo, underscoring extracellular Wnt cues as important brakes on osteoclast activity [33]. circFAM190a, a circular RNA upregulated during osteoclastogenesis, promotes osteoclast formation and bone loss in vivo via Akt1 stabilization (HSP90β-dependent), highlighting non-coding RNA programs that potentiate osteoclastogenic signaling [34]. Among natural products, icaritin derivatives inhibit RANKL-induced osteoclastogenesis through TRAF6–NF-κB/MAPK pathways [35], and micheliolide prevents estrogen-deficiency bone loss by suppressing p38 MAPK/NF-κB signaling during osteoclast differentiation [36]. In this landscape, SWS1 represents a distinct intracellular Ca^2+^-binding adaptor that concurrently attenuates proximal RANKL signaling and cytoskeleton-coupled co-stimulatory pathways, thereby integrating signal transduction with actin-ring/podosome organization. These comparisons position SWS1 as complementary to extracellular Wnt modulators, non-coding RNA effectors, and small-molecule inhibitors, and may guide combination strategies targeting both transcriptional and cytoskeletal nodes of osteoclast function.

Our in vivo findings corroborate the cellular data: SWS1 KO mice displayed significant trabecular bone loss, increased TRAP-positive osteoclasts, and elevated osteoclast surface area, all indicative of increased osteoclast activity. By contrast, baseline osteoblast metrics in the distal femoral metaphysis were unchanged between WT and SWS1-KO mice. Quantitative histomorphometry revealed comparable Ob.S/BS and N.Ob/BS across genotypes (Figure 3D), in agreement with unaltered ALP/ARS mineralization in primary osteoblast cultures and similar von Kossa staining of femora (Figure S7). These observations suggest minimal osteoblast-related side effects under homeostatic conditions, whereas SWS1 primarily constrains the osteoclast axis in vivo. These results position SWS1 as a physiological suppressor of osteoclast function and skeletal remodeling. Interestingly, unlike other known inhibitors of osteoclastogenesis such as osteoprotegerin or IRF8, SWS1 appears to act both at the transcriptional and cytoskeletal levels, integrating signal transduction and cellular architecture [37].

We also acknowledge that bone formation depends on skeletal stem cells (SSCs) that supply osteoblasts. A rigorous test of SWS1 effects on SSC pool size and bone-forming output would require prospective SSC isolation with validated marker sets, clonogenic assays, and transplantation/lineage-tracing in defined skeletal sites—beyond the scope of this osteoclast-focused study. Notably, recent work emphasizes SSCs as functionally defined and therapeutically promising populations with greater definitional precision than historically heterogeneous “MSCs” [38,39]. Future studies will determine whether SWS1 modulates SSC dynamics or osteoprogenitor coupling in the bone remodeling unit.

Taken together, this study identifies SWS1 as a multifunctional inhibitor of osteoclastogenesis. It attenuates early signaling cascades, limits the induction arm of the Ca^2+^/NFATc1 program, and constrains cytoskeletal signaling required for bone resorption. These findings not only reveal a new layer of intracellular control in osteoclast differentiation but also raise the possibility that SWS1 could serve as a biomarker or therapeutic target in bone diseases characterized by excessive resorption. Future work should focus on defining the direct molecular interactions of SWS1 in osteoclasts and evaluating its expression and function in disease contexts such as osteoporosis, rheumatoid arthritis, and cancer-associated bone loss. Additionally, it will be important to determine whether SWS1 expression is modulated by systemic factors or local inflammatory signals, which could provide insight into its regulation and potential for therapeutic intervention.

## 4. Materials and Methods

### 4.1. Materials

Recombinant human M-CSF and RANKL were obtained from PeproTech (London, UK). Primary antibodies against SWS1 (sc-292051), NFATc1 (sc-7294, 7A6), PLCγ2 (sc-5283, B-10), and IκB (sc-371, C-21) (Santa Cruz Biotechnology, Santa Cruz, CA, USA) were used. Additional antibodies recognizing c-Fos (sc-166940, E-8), Akt (#9272), p-Akt (#9271), p38 (#9212), p-p38 (#9211), JNK (#9252), p-JNK (#9251), ERK (#9102), p-ERK (#9101), p-IκB (#2859, 14D4), BTK (#3533, C82B8), p-PLCγ2 (#3874), cortactin (#3502), p-cortactin (#4569), Src (#2123, 32G6), p-Src (#6943, D49G4), p-Syk (#2710, C87C1), Cbl (#2747), and p-Cbl (#3555) were supplied by Cell Signaling Technology (Beverly, MA, USA). The p-Btk (GTX61791, EP420Y) came from GeneTex (Irvine, CA, USA), while β-actin (A5441, AC-15) was obtained from Sigma-Aldrich (St. Louis, MO, USA). HRP-conjugated anti-rabbit and anti-mouse secondary antibodies were sourced from Enzo Life Sciences (Farmingdale, NY, USA).

### 4.2. Animal Experiments

All animal procedures complied with the Institutional Animal Care and Use Committee (IACUC) of Wonkwang University and were approved under protocol WKU18-05. SWS1 KO mice were kindly provided by Dr. Chang-Duk Jun of the Gwangju Institute of Science and Technology. Animals were maintained under controlled environmental conditions (22–24 °C, 55–60% humidity, 12 h light/dark cycle) with free access to water and a standard irradiated chow diet (SAM #31; Samtako Bio-Korea Inc., Osan, Republic of Korea). General health was monitored throughout the study by recording body weight and behavioral status, and only mice without apparent abnormalities were included in subsequent analyses. 

### 4.3. Cell Culture

Mouse BMM cells were isolated from the femurs and tibias of 5-week-old male SWS1 WT, KO, or ICR mice. Bone marrow progenitors from mice were transformed into macrophages upon exposure to M-CSF (30 ng/mL) for 3 days. BMMs were seeded at a density of 3.5 × 10^4^ cells in 48-well plates or 3 × 10^5^ cells in 6-well plates for osteoclast differentiation. Osteoclast formation was achieved by treating mouse BMMs with 30 ng/mL M-CSF and 100 ng/mL RANKL for 3 days.

### 4.4. Cell Proliferation Assay

BMM cells (1 × 10^4^ cells) were cultured in 96-well plates with 10% α-MEM in the presence of M-CSF (30 ng/mL) and each concentration of SWS1 RP. BMM cells grown in a cell culture plate were incubated with the XTT labeling mixture for 4 h. The cells were quantified using a scanning ELISA reader at 450 nm.

### 4.5. F-Actin Ring Formation and Immunofluorescence

BMMs (3.5 × 10^4^ cells) were seeded into 48-well plates and cultured with M-CSF (30 ng/mL) and RANKL (100 ng/mL) for three days to induce osteoclast differentiation. Cells were then fixed with 3.7% formaldehyde prepared in phosphate-buffered saline (PBS) for 20 min, followed by permeabilization with 0.1% Triton X-100 for 10 min. After rinsing with PBS, samples were blocked in 2% bovine serum albumin for 2 h and incubated overnight with anti-SWS1. Actin filaments were visualized with Alexa Fluor^TM^ 568-conjugated phalloidin (Thermo Fisher Scientific, San Diego, CA, USA) for 10 min, and nuclei were counterstained with 4′, 6-diamidino-2-phenylindole dihydrochloride (DAPI) for 1 min. Alexa Fluor^TM^ 488-labeled secondary antibody was applied to detect the anti-SWS1. Fluorescence images were captured using a Ts2 microscope (Nikon, Shinagawaku, Tokyo, Japan).

### 4.6. Bone Resorption Assay

Primary osteoblasts (1 × 10^6^ cells) and BMCs (1 × 10^7^ cells) were co-cultured for 7 days on collagen gel-coated dishes in the presence of 1 × 10^−8^ M 1,25-dihydroxyvitamin D_3_ (VitD_3_) and 1 × 10^6^ M prostaglandin E_2_ (PGE_2_) (Sigma-Aldrich). Mature osteoclasts generated from this co-culture were released with 0.1% collagenase for 10 min at 37 °C and subsequently seeded onto dentin slices or hydroxyapatite-coated plates (Corning, Corning, NY, USA). After incubation for 24–48 h, cells were removed, and resorption lacunae were visualized under microscopy. The excavated pits were then stained with hematoxylin solution and quantified using ImageJ 1.54k software (NIH, Bethesda, MD, USA).

### 4.7. Quantitative Real-Time PCR (qRT-PCR)

First-strand cDNA was generated from 2 μg of total RNA using SuperScript II Reverse Transcriptase (Thermo Fisher Scientific, Vilnius, Lithuania). qRT-PCR was performed with Accupower green star qRT-PCR master mix (Bioneer, Daejeon, Republic of Korea) on an ExcyclerTM 96 Real-Time thermal cycler. Primer sequences for the analyzed genes were as follows: mus*GAPDH* forward: 5′-TCAAGAAGGTGGTGAAGCAG-3′; reverse: 5′-AGTGGGAGTTGCTGTTGAAGT-3′; mus*SWS1* forward: 5′-TCGACCTGATGGAGCTGAAACTCA-3′; reverse: 5′-ACACGTTGATGGCCTGTACCTT-3′; mus*OSCAR* forward: 5′-GGAATGGTCCTCATCTCCTT-3′; reverse: 5′-TCCAGGCAGTCTCTTCAGTTT-3′; mus*αv-integrin*: forward: 5′-ACAAGCTCACTCCCATCACC-3′; reverse: 5′-ATATGAGCCTGCCGACTGAC-3′; mus*β3-integrin*: forward: 5′-GGAGTGGCTGATCCAGATGT-3′; reverse: 5′-TCTGACCATCTTCCCTGTCC-3′; mus*c-Fos* forward: 5′-AGTCCATTTGCTGACCCCAC-3′; reverse: 5′-GGATGGTCGTGTTGATGCG-3′; mus*NFATc1* forward: 5′-GAGTACACCTTCCAGCACCTT-3′; reverse: 5′-TATGATGTCGGGGAAAGAGA-3′; mus*Atp6v0d2* forward: 5′-GACCCTGTGGCACTTTTTGT-3′; reverse: 5′-GTGTTTGAGCTTGGGGAGAA-3′; mus*DC-STAMP* forward: 5′- TCCTCCATGAACAAACAGTTCCA-3′; reverse: 5′-AGACGTGGTTTAGGAATGCAGCTC-3′; mus*Ctsk* forward: 5′-CCAGTGGGAGCTATGGAAGA-3′; reverse: 5′-CTCCAGGTTATGGGCAGAGA-3′; mus*Cortactin* forward: 5′-GCAAGTTCGGTGTCCAGATG-3′; reverse: 5′-CACGGCACTCTTGTCTACAC-3′. The cycling program consisted of an initial denaturation at 95 °C for 5 min, followed by 40 cycles of 95 °C for 1 min, 60 °C for 30 s, and 72 °C for 1 min. *GAPDH* served as the internal control. Relative transcript levels were expressed as fold changes and calculated using the 2^−ΔΔCt^ method.

### 4.8. Western Blotting

Following treatments, cells were washed with cold PBS and lysed in lysis buffer composed of 50 mM Tris-HCl, 150 mM NaCl, 5 mM EDTA, 1% Triton X-100, 1 mM sodium vanadate, 1% deoxycholate, and protease inhibitors. Protein concentration was assessed using the DC Protein Assay Kit (Bio-Rad Laboratories Inc., Hercules, CA, USA). Equivalent protein samples (20–30 μg) were resolved on an 8–12% SDS-PAGE gel and subsequently transferred to polyvinylidene difluoride membranes (Millipore, Bedford, MA, USA). Membranes were blocked in 5% nonfat milk prepared in Tris-buffered saline with 0.1% Tween 20 (TBST) for 1 h, rinsed, and incubated with primary antibodies for 3 h, followed by incubation with secondary antibodies for 2 h. After additional washes in TBST, signals were visualized with Immobilon Western Chemiluminescent HRP Substrate (Millipore, Billerica, MA, USA).

### 4.9. siRNA Transfection

siRNA control and siSWS1 were designed and synthesized by Santa Cruz Biotechnology. The siRNAs were transfected into BMMs using Lipofectamine 3000^TM^ reagent (Thermo Fisher Scientific). Briefly, after culturing BMMs in α-MEM containing 10% FBS and lacking antibiotics for 2 days, each siRNA (10 nM) and Lipofectamine 3000 (0.5 mL/48-well, 3 mL/6-well) mixture in a serum-reduced Opti-MEM^TM^ medium (Thermo Fisher Scientific) was added to BMMs and incubated for 6 h. A new differentiation induction medium (10% α-MEM) was added, and the cells were further differentiated into osteoclasts.

### 4.10. Micro-Computed Tomography (Micro-CT)

Micro-CT was carried out on excised femurs using a volumetric scanner (NFR-Polaris-G90; NanoFocusRay, Jeonju, Republic of Korea). Scanning was performed at 65 kV, 90 μA, with an exposure time of 313 ms/frame across 512 projections. Image reconstruction produced datasets of 1024 × 1024 pixels over 512 consecutive slices, stored in DICOM format. Structural indices obtained included total volume (TV; mm^3^), bone volume (BV; mm^3^), bone volume/total volume (BV/TV; %), bone mineral density (BMD; g/cm^2^), bone surface (mm^2^), trabecular number (1/mm), trabecular thickness (mm), and trabecular separation (mm).

### 4.11. Histological Staining

Paraffin sections were cut at 5 μm thickness using an automated system for tissue sectioning and staining (Leica, HistoCore AUTOCUT) at the Core Facility for Supporting Analysis & Imaging of Biomedical Materials at Wonkwang University, supported by the National Research Facilities and Equipment Center. The sections were then stained with hematoxylin (1.5 min)/eosin (45 s) or TRAP solution (30 min), dipped one by one in xylene, and mounted with a mounting medium. The number of osteoclasts present in mouse femurs was determined by histomorphometric analysis of TRAP-stained sections. Stained bones were imaged using a Nikon Ts2 microscope for TRAP-stained osteoclasts and eroded bone surface images.

### 4.12. Statistical Analysis

Each experiment was independently repeated at least three times, and results are reported as the mean values with standard deviation. Group differences were analyzed by one-way ANOVA with Tukey’s post hoc test or Student’s t-test where appropriate. Statistical significance was defined as *p*-value *<* 0.05.

## 5. Conclusions

Swiprosin-1 suppresses osteoclast differentiation and bone resorption by inhibiting RANKL-induced signaling pathways and cytoskeletal remodeling. Its deficiency enhances osteoclastogenic transcriptional activation and actin ring formation through upregulation of MAPK, NF-κB, and ITAM-associated signaling. These findings establish Swiprosin-1 as a key intracellular regulator of osteoclast function and bone homeostasis.

## Data Availability

Data will be made available on request.

## Supplementary Materials

The following supporting information can be downloaded at: https://www.mdpi.com/article/10.3390/ijms26178613/s1.

## Abbreviations

The following abbreviations are used in this manuscript:

SWS1	Swiprosin-1
KO	Knockout
NF-κB	Nuclear factor-kappa B
RANK	Receptor activator of NF-κB
TRAFs	Tumor necrosis factor receptor-associated factors
MAPKs	Mitogen-activated protein kinases
NFATc1	Nuclear factor of activated T cells cytoplasmic 1
OSCAR	Osteoclast-associated receptor
Atp6v0d2	v-ATPase subunit d2
Ctsk	Cathepsin K
EFhd2	EF-hand domain-containing protein 2
LPS	Lipopolysaccharide
SH3	Src homology 3
MNCs	Multinucleated cells
BV	Bone volume
Tb.Sp	Trabecular separation
Tb.Th	Trabecular thickness
Tb.N	Trabecular number
BMD	Bone mineral density
Oc.S/BS	Osteoclast surface per bone surface
M-CSF	Macrophage colony-stimulating factor
IACUC	Institutional Animal Care and Use Committee
BMM	Bone marrow-derived macrophage
WT	Wild-type
DAPI	4′, 6-diamidino-2-phenylindole dihydrochloride
VitD_3_	1,25-dihydroxyvitamin D_3_
PGE_2_	Prostaglandin E_2_
qRT-PCR	Quantitative Real Time-PCR
PBS	Phosphate-buffered saline
TBST	Tris-buffered saline with 0.1% Tween 20
siRNA	Small interfering RNA
siSWS1	siRNA for SWS1
Micro-CT	Micro-computed tomography
