# Supplementary material for: Swiprosin-1 Negatively Regulates Osteoclast Differentiation and Bone Resorption via Akt/MAPK/NF-κB Pathway and αvβ3 Integrin-Dependent Signaling
Source: Int J Mol Sci. 2025 Sep 4;26(17):8613. doi: 10.3390/ijms26178613 (PMC12429813; doi:10.3390/ijms26178613)
Supplement: Supplementary file 1 [file ijms-26-08613-s001.zip › ijms-3832723-supplementary.pdf]

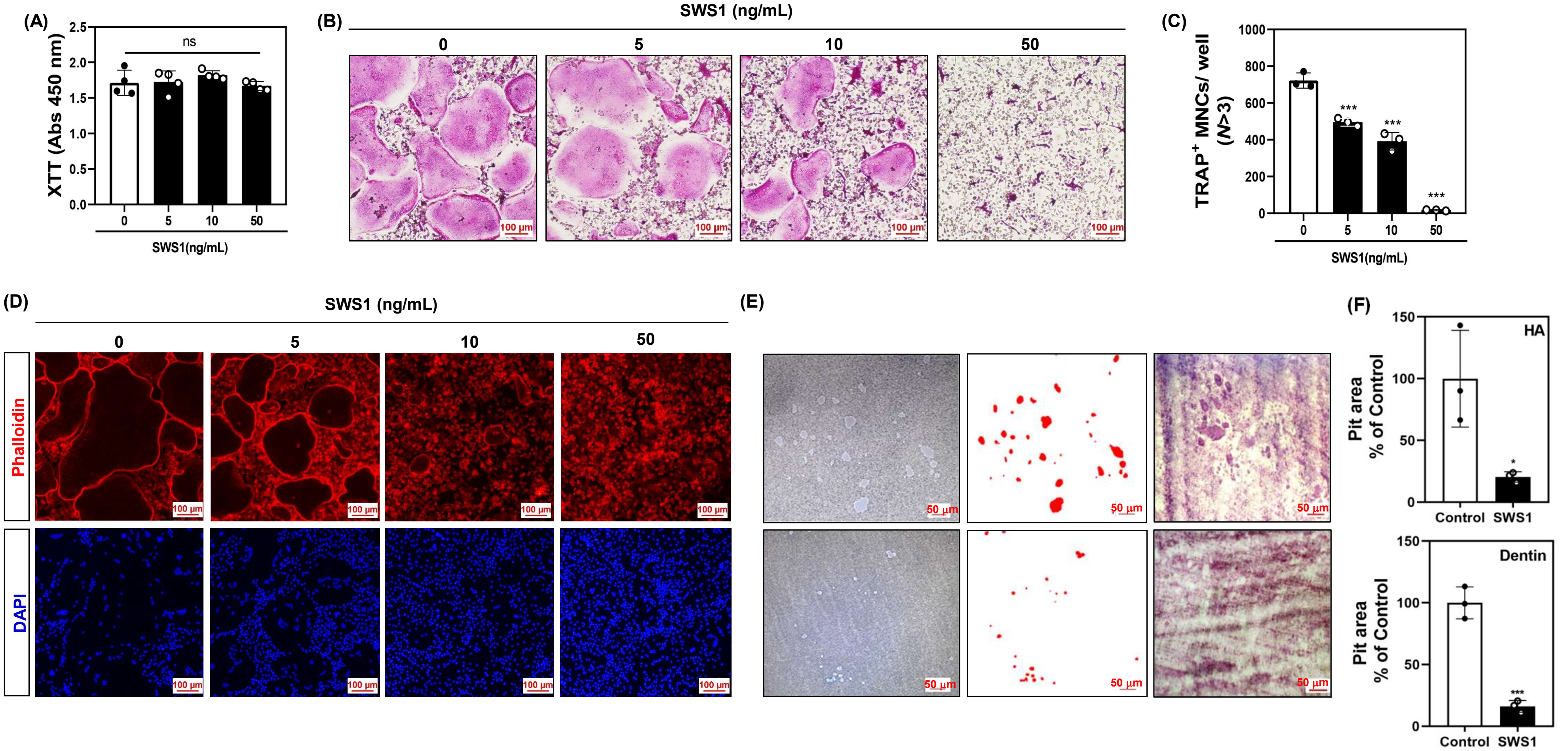

Figure S1.

Effect of recombinant SWS1 protein on osteoclast differentiation, F-actin ring formation, and bone resorption in vitro. (A) Cell viability of mouse BMMs treated with various concentrations of SWS1 (0, 5, 10, 50 ng/mL) as measured by XTT assay. (B) Representative images of TRAP-stained multinucleated osteoclasts (MNCs) formed after RANKL stimulation with or without SWS1 treatment. (C) Quantification of TRAP$^+$ MNCs (≥3 nuclei) per well. (D) Representative confocal microscopy images of F-actin rings (phalloidin staining, red) and nuclei (DAPI, blue) in osteoclasts treated with SWS1 at indicated concentrations. (E) Representative bone resorption pit images on hydroxyapatite (HA)-coating plate or dentine slices treated with SWS1, with visualized resorption areas in binary contrast. (F) Quantification of total resorption area on HA or dentine slices.

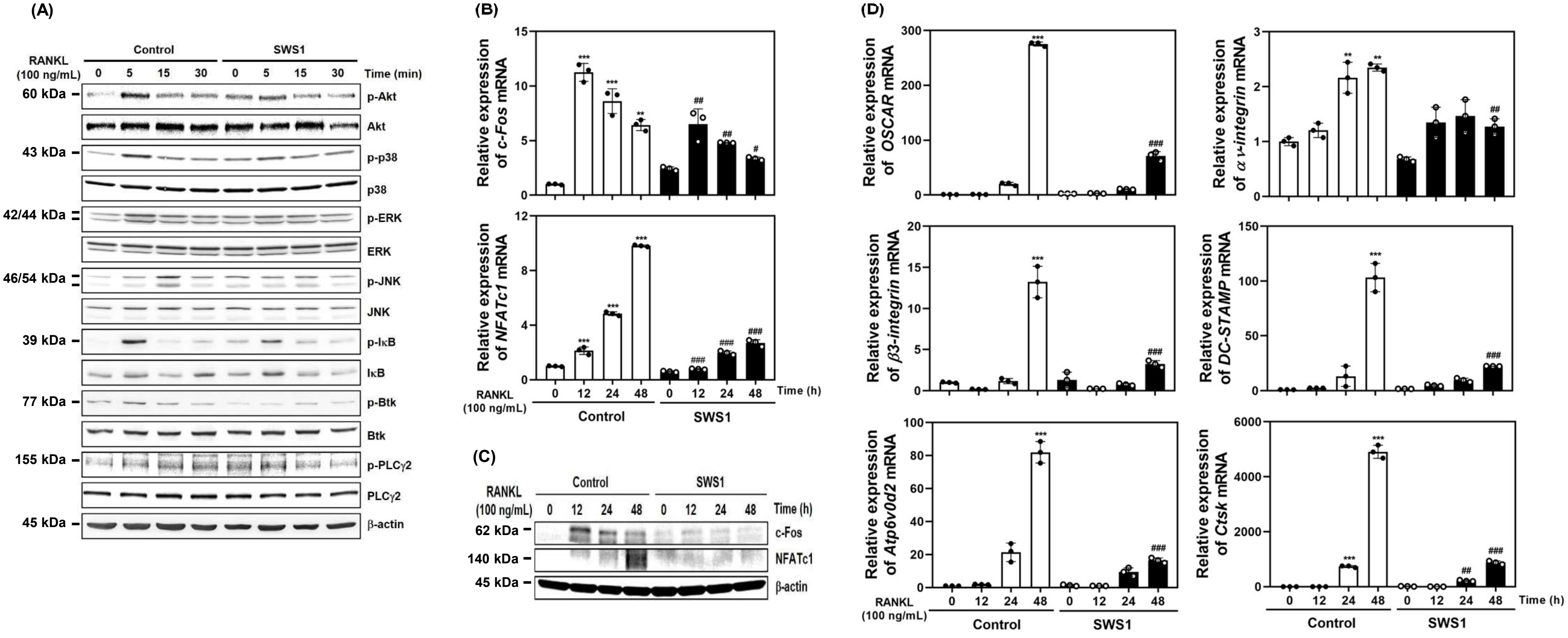

Figure S2.

Effect of recombinant SWS1 protein on RANKL-induced intracellular signaling and osteoclastogenic transcription factor expression. (A) Time-dependent phosphorylation of Akt, p38, ERK, JNK, IκB, Btk, and PLCγ2 in RANKL-treated BMMs with or without SWS1 treatment, analyzed by Western blotting. (B) qRT-PCR analysis of c-Fos and NFATc1 mRNA expression in RANKL-stimulated BMMs with or without SWS1 treatment. (C) Time-course Western blot analysis of c-Fos and NFATc1 expression in RANKL-stimulated BMMs with or without SWS1 treatment. (D–E) Expression of osteoclast-related genes including SWS1, OSCAR, αυ-integrin, β3-integrin, DC-STAMP, Atp6v0d2, and Ctsk. The mRNA expression was normalized with *GAPDH*. Data are presented as the mean ± SD of three independent experiments. $^{**}p$<0.01; $^{***}p$<0.001 versus Control cells. $^{\#}p$<0.05; $^{\#\#}p$<0.01; $^{\#\#\#}p$<0.001 versus the Control cells at the indicated time point.

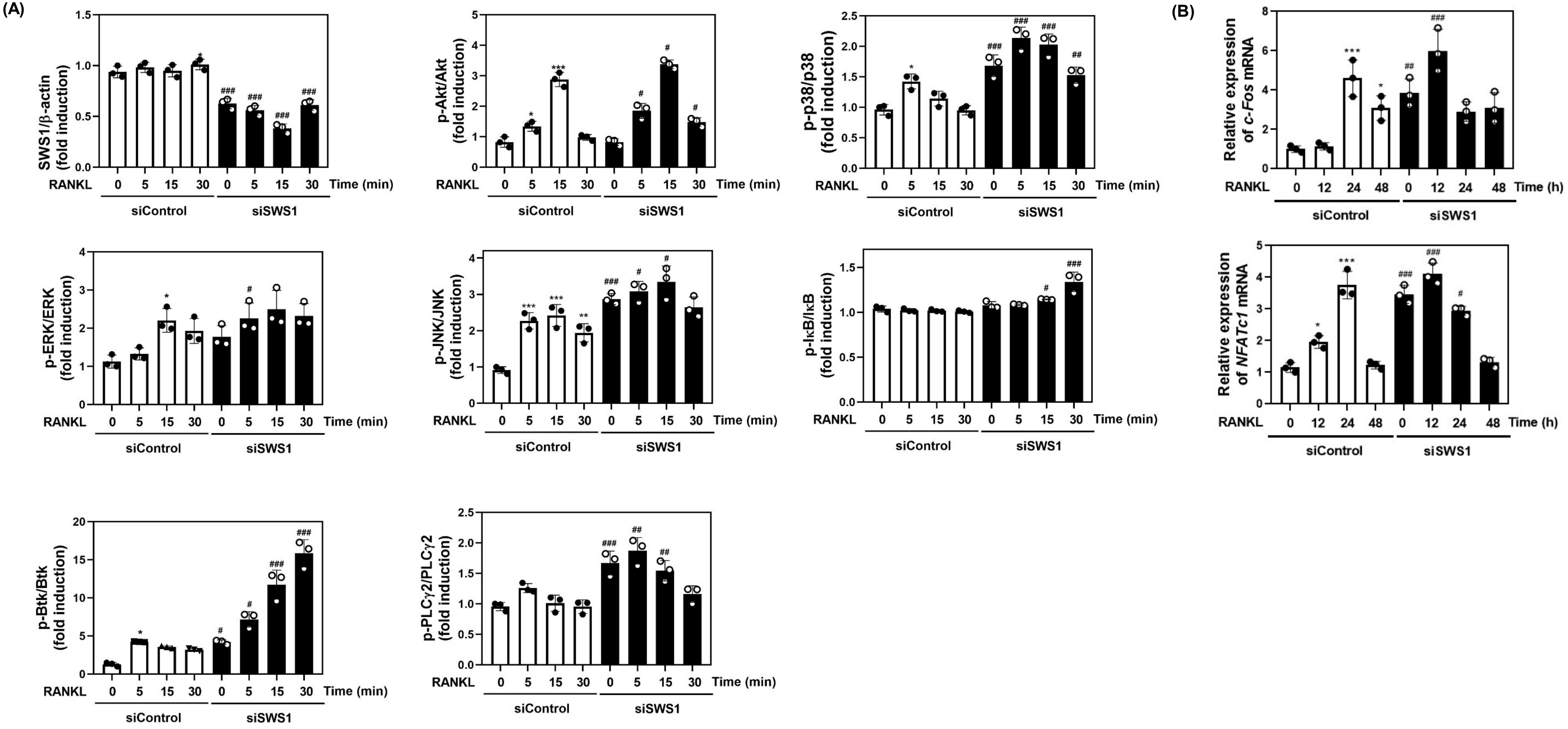

Figure S3.

Quantification of Western blot analysis shown in Figure 2. (A and B) BMMs were transfected with control siRNA (siControl) or siRNA targeting Swiprosin-1 (siSWS1) and then stimulated with RANKL for the indicated times. Relative band intensities of Western blot data in Figure 2 were quantified and normalized to internal controls. Data are presented as the mean ± SD of three independent experiments. $^{*}p<0.05$; $^{**}p<0.01$ versus Control cells. $^{\#}p<0.05$; $^{\#\#}p<0.01$; $^{\#\#\#}p<0.001$ versus the siControl cells at the indicated time point.

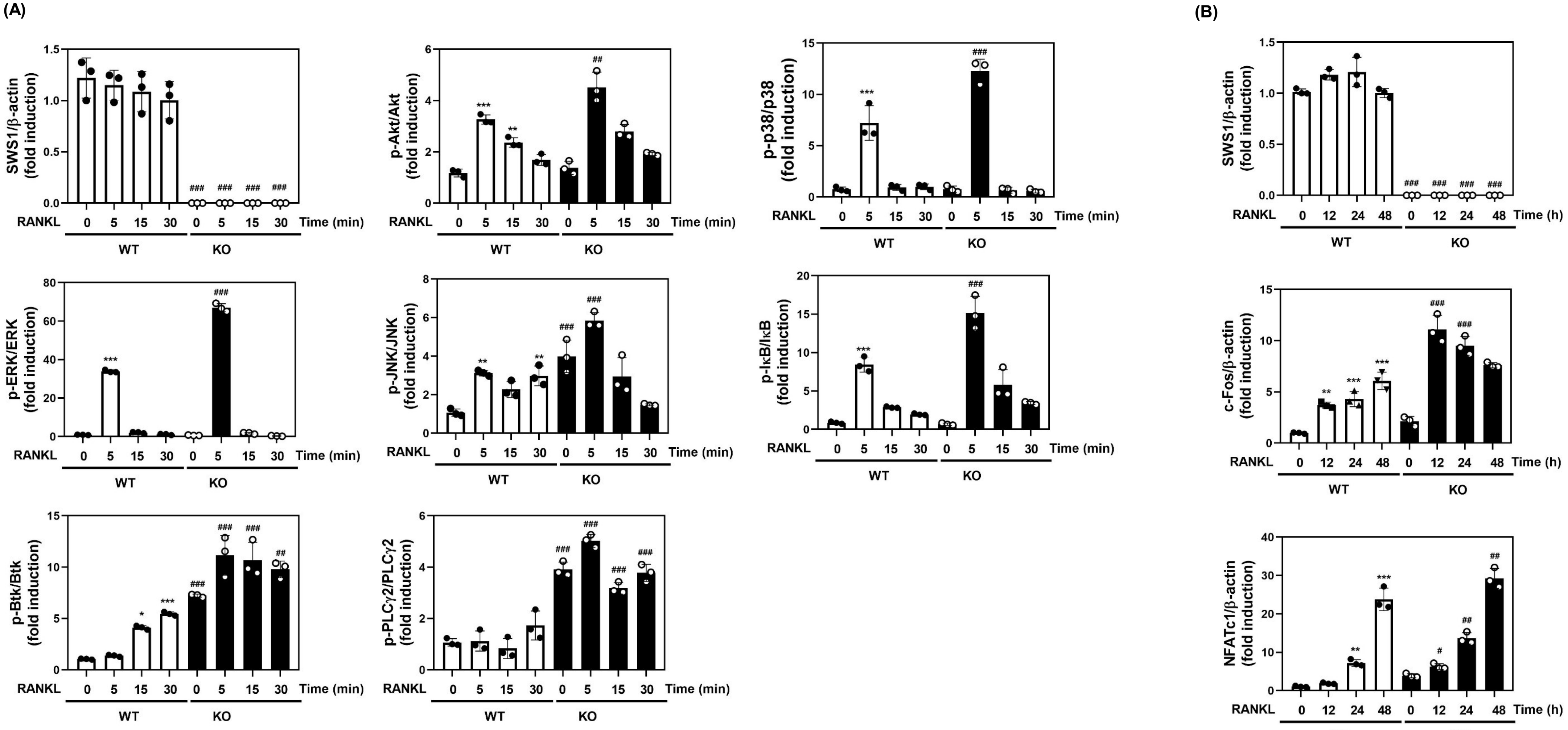

Figure S4.
Quantification of Western blot analysis shown in Figure 5. (A and B) BMMs from wild-type (WT) and SWS1 knockout (KO) mice were stimulated with RANKL for the indicated times. Relative band intensities of Western blot results in Figure 5 were quantified and normalized to internal controls. Data are presented as the mean ± SD of three independent experiments. $^{*}p<0.05$; $^{**}p<0.01$; $^{***}p<0.001$ versus Control cells. $^{\#}p<0.05$; $^{\#\#}p<0.01$; $^{\#\#\#}p<0.001$ versus the WT cells at the indicated time point.

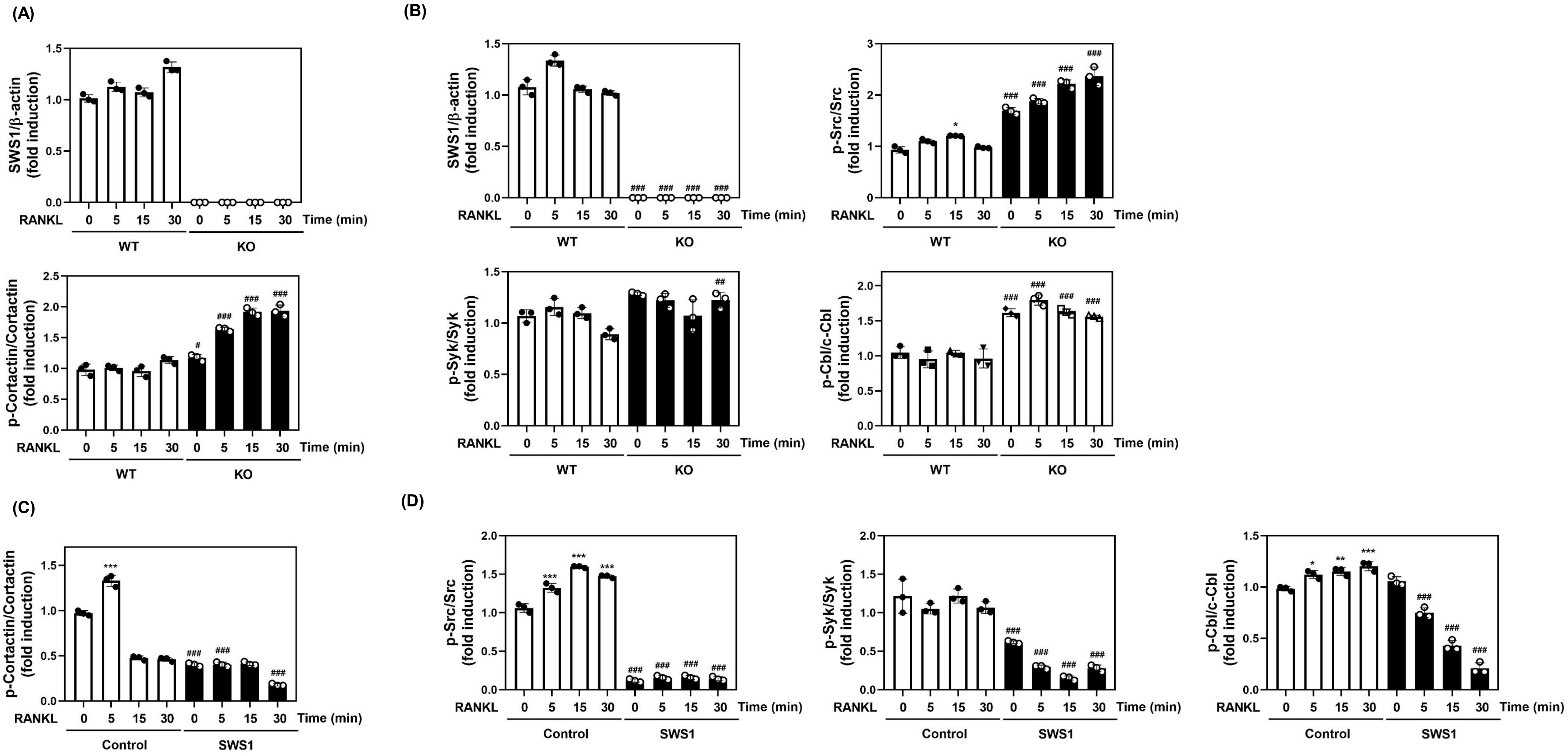

Figure S5.

Quantification of Western blot analysis shown in Figure 6. BMMs from wild-type (WT) and SWS11 knockout (KO) mice, or BMMs treated with recombinant SWS1 protein (SWS1), were stimulated with RANKL for the indicated times. Relative band intensities of Western blot results in Figure 6 were quantified and normalized to internal controls. Data are presented as the mean ± SD of three independent experiments. $^{*}p<0.05$; $^{**}p<0.01$; $^{***}p<0.001$ versus Control cells. $^{\#}p<0.05$; $^{\#\#}p<0.01$; $^{\#\#\#}p<0.001$ versus the WT or Control cells at the indicated time point.

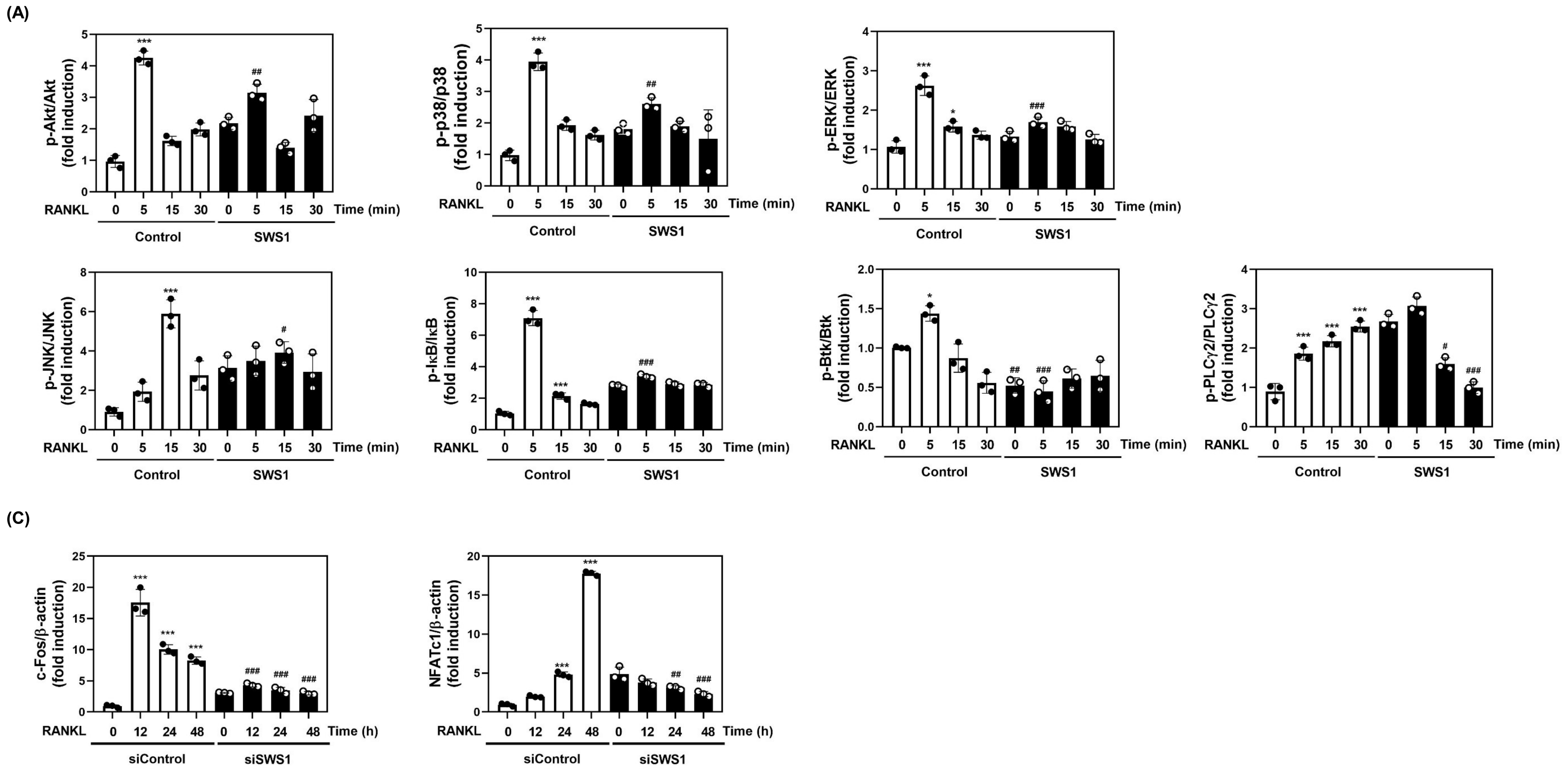

Figure S6.

Quantification of Western blot analysis shown in Figure S2. BMMs were treated with recombinant SWS1 protein (SWS1) and stimulated with RANKL for the indicated times. Relative band intensities of Western blot results in Figure S2 were quantified and normalized to internal controls. Data are presented as the mean ± SD of three independent experiments. $^{*}p$<0.05; $^{**}p$<0.01; $^{***}p$<0.001 versus Control cells. $^{\#}p$<0.05; $^{\#\#}p$<0.01; $^{\#\#\#}p$<0.001 versus the Control cells at the indicated time point.

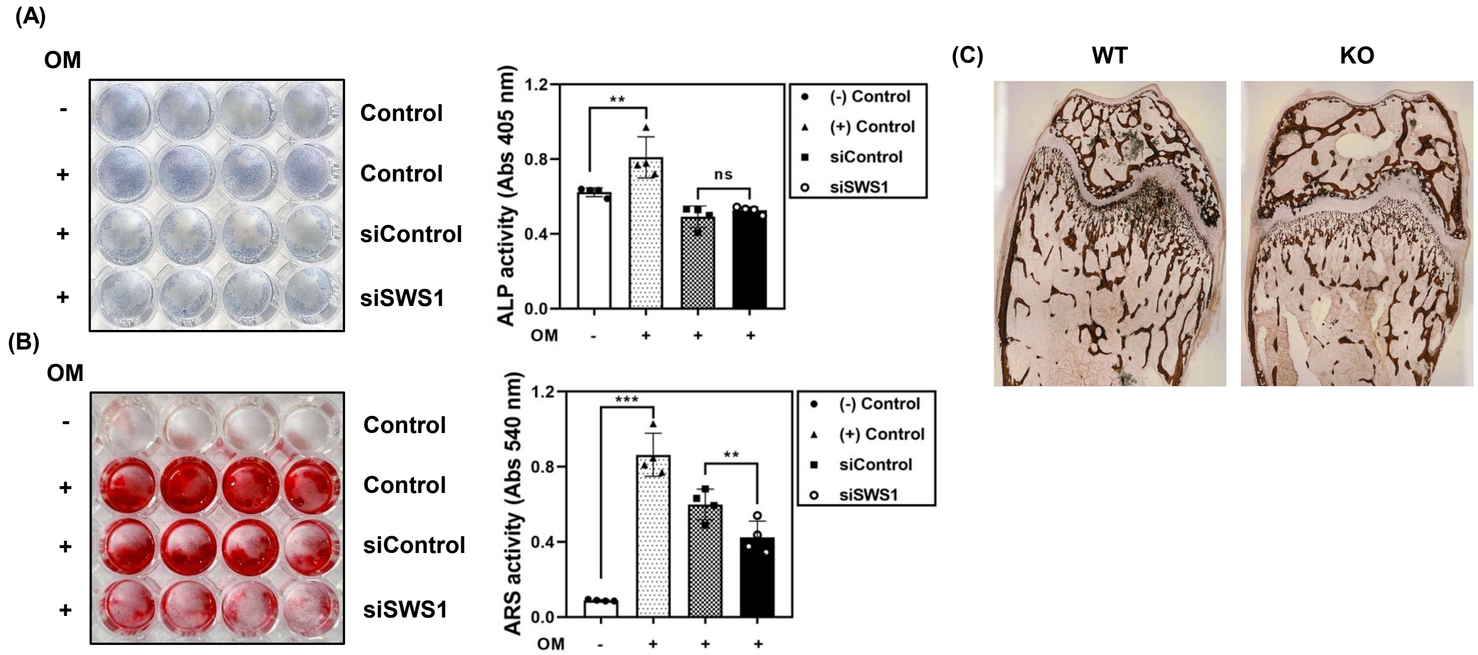

Figure S7.

Effects of Swiprosin-1 silencing and deficiency on osteoblast differentiation and bone mineralization. (A) Representative images of ALP staining (left) and quantification of ALP activity (Abs 405 nm, right) in osteogenic medium (OM) with or without siSWS1 treatment. (B) Representative images of Alizarin Red S (ARS) staining (left) and quantification of ARS activity (Abs 540 nm, right) under the same conditions. Data are presented as mean ± SD. $^{**}p < 0.01$, $^{***}p < 0.001$, ns: not significant. (C) von Kossa staining of femoral sections from SWS1 wild-type (WT) and knockout (KO) mice, showing differences in mineral deposition and trabecular architecture.
